# RNA-sequencing in ophthalmology research: considerations for experimental design and analysis

**DOI:** 10.1177/2515841419835460

**Published:** 2019-03-15

**Authors:** Nicholas Owen, Mariya Moosajee

**Affiliations:** Development, Ageing and Disease Theme, UCL Institute of Ophthalmology, University College London, London, UK; Development, Ageing and Disease Theme, UCL Institute of Ophthalmology, University College London, London, UK; NIHR Biomedical Research Centre for Ophthalmology, Moorfields Eye Hospital NHS Foundation Trust, London, UK; Great Ormond Street Hospital for Children NHS Foundation Trust, London, UK

**Keywords:** bioinformatics, differential gene expression, false discovery rate, gene ontology, next-generation sequencing, ophthalmology, power, replicates, RNA-sequencing, transcriptomics

## Abstract

High-throughput, massively parallel sequence analysis has revolutionized the way that researchers design and execute scientific investigations. Vast amounts of sequence data can be generated in short periods of time. Regarding ophthalmology and vision research, extensive interrogation of patient samples for underlying causative DNA mutations has resulted in the discovery of many new genes relevant to eye disease. However, such analysis remains functionally limited. RNA-sequencing accurately snapshots thousands of genes, capturing many subtypes of RNA molecules, and has become the gold standard for transcriptome gene expression quantification. RNA-sequencing has the potential to advance our understanding of eye development and disease; it can reveal new candidates to improve our molecular diagnosis rates and highlight therapeutic targets for intervention. But with a wide range of applications, the design of such experiments can be problematic, no single optimal pipeline exists, and therefore, several considerations must be undertaken for optimal study design. We review the key steps involved in RNA-sequencing experimental design and the downstream bioinformatic pipelines used for differential gene expression. We provide guidance on the application of RNA-sequencing to ophthalmology and sources of open-access eye-related data sets.

## Introduction

With the advent of high-throughput sequencing technologies, focus on temporal gene expression through examination of the active transcriptome of tissues, cells, and model systems using RNA-sequencing (RNA-seq) has increased.^1^ In ophthalmology and vision research, RNA-seq utilization is extensive. For example, investigation of gene expression changes in corneal epithelial tissue from keratoconus patients has provided insights into the cause of this progressive corneal degeneration.^2^ Pathways including Wnt, Hedgehog, and Notch1 signaling were shown to be significantly reduced in keratoconus epithelium. In glaucoma, the leading cause of irreversible blindness worldwide characterized by the progressive loss of retinal ganglion cells (RGCs),^3,4^ investigations into the RGC transcriptome of induced pluripotent stem cells (iPSCs) from patients with the *SIX6* risk allele [missense variant rs33912345; C>A; p.(His141Asn)] associated with reduced retinal nerve fiber layer thickness, and mouse models of optic nerve head damage have identified critical pathophysiologic pathways, such as endoplasmic reticulum stress, Notch signaling, and mammalian target of rapamycin (mTOR) pathway.^5–7^ Elucidation of transcript signatures in lens development has revealed the expression of novel transcripts decreasing in postnatal tissue.^8^ Lens-enriched expression analysis has confirmed high expression of established cataract-linked genes, such as the *Crystallin* gene family, and identified a number of transcription factors as novel potential regulators in the lens.^9^ RNA-seq of rod photoreceptors from the zebrafish has identified novel expression of genes not previously thought to be expressed in this cell type including *opsin 4.1* and several nuclear hormone receptor genes.^10^ Similar experiments on dissociated mouse cones have provided an insight into the gene expression patterns occurring throughout postnatal development, highlighting 14% of all genes detected were switched off around postnatal day 6 (P6), including those encoding transcription factors, neurogenesis, and cone-specific genes.^11^ Such investigations reveal the role of previously unknown or unclassified transcripts in eye development, for example, the characterization of zebrafish *zic2*, which restricts *pax2a* expression and Hedgehog signaling, when ablated causes chorioretinal coloboma^12^ and identification of numerous miRNAs regulating pathways not previously associated with retinal degeneration, using retinal pigment epithelium (RPE) cells under oxidative stress as a model system.^13^ In this manner, novel information is gleaned; new targets for potential molecular diagnosis or therapeutic interventions may emerge.^14,15^ In this review, we will cover the considerations for the design and execution of a typical RNA-seq project investigating differentially expressed messenger RNA (mRNA). We will provide recent examples of the utilization of RNA-seq within the field of ophthalmology.

## Considerations for RNA-seq experimental design

With no single optimal pipeline for this experimentation, combined with no standard application and analysis approach, the use of RNA-seq data can be daunting. Experimental plan and strategic approaches depend highly on the type of RNA and or organism being studied, as well as the goals of the research. One may utilize previously reported species transcriptomes to guide the alignment of reads or align without prior knowledge to identify potentially novel transcripts.

**Figure 1.**
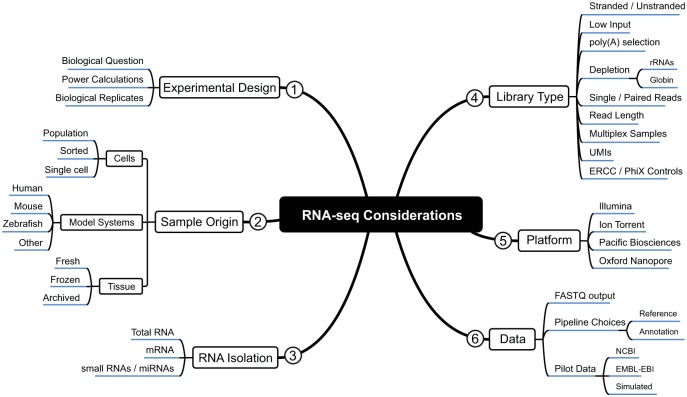
A diagrammatic overview of the considerations for designing a successful RNA-seq experiment for differential gene expression analysis. Branches of the outline are numbered to indicate the general order for the considerations. Within each branch, subbranches denote options to consider within the design.

One of the most crucial requirements for a successful RNA-seq experiment is the biological question of interest and how the data generated can answer that. Figure 1 summarizes critical aspects for an optimal experimental design. Number of sample replicates is of importance as increasing the number per biological condition has a more significant impact on the accuracy of the data produced over increasing sequencing depth.^16,17^ A growing number of algorithms can calculate the required sample number for significance and power of experiments; including Scotty,^18^ powsimR,^19^ PROPER,^20^ and RNASeqPower.^21^ Technical replicates are generally not required for differential expression analysis, as RNA-seq has been shown to be accurate as well as reproducible.^22–24^

For pilot studies to assess accuracy and variance of analysis at different stages of an RNA-seq pipeline, simulated data can be created through synthetic reads generated from genomic sequence.^25,26^ It is also possible to utilize transcriptomic data submitted to public repositories, such as EMBL ENA^27^ and National Center for Biotechnology Information (NCBI) SRA,^28^ to obtain information on the variance of data. Table 1 summarizes the current obtainable experimental RNA-seq data sets related to ophthalmology and vision research at NCBI. Combination of data with published data sets from different biological samples, sequencing centers, or varying experimental protocols may lead to incorporation of batch effects. Such meta-analysis, therefore, would have decreased statistical power and accuracy, even in well-designed studies.^54^ A significant source of false discovery of differential expression is commonly across batches of experiments rather than across the biological groups of interest.^55^

**Table 1.** Summary of a subset of NCBI-submitted RNA-seq experimental data sets related to eye development and disease, highlighting utilized methods and software. The NCBI Gene Expression Omnibus (GEO) was searched for terms ‘retina disease; retina development; eye disease; eye development’, subsetting on ‘Study type’ – ‘expression profiling by high throughput sequencing’ (December 2018) (available details of the software used for analysis are noted; *unpublished data sets).

Keywords	Data set description	Species	NCBI GEO	Software	Reference
Cornea	Molecular Effects of Doxycycline Treatment on Pterygium as Revealed by Massive Transcriptome Sequencing	*Homo sapiens*	GSE34736	Tuxedo: TopHat2, Cufflinks2	Larrayoz and colleagues^29^
Cornea	RNA-seq analysis in Cornea epithelial cells (CECs), skin epithelial cells (SECs), LSCs after knocking down PAX6 (3-D shPAX6 LSCs) and SESCs transduced with PAX6 (3-D PAX6+SESCs) upon 3-D differentiation	*H. sapiens*	GSE54322	Not reported	Ouyang and colleagues^30^
Cornea	Molecular Effects of Doxycycline Treatment on Pterygium from Caucasian Patients as Revealed by Massive Transcriptome Sequencing	*H. sapiens*	GSE58441	Tuxedo: TopHat2, Cufflinks2	Larrayoz and colleagues^29^
Cornea	RNA-seq analysis and comparison of corneal epithelium in keratoconus and myopia patients	*H. sapiens*	GSE112155	TopHat2, edgeR, DESeq2, limma	You and colleagues^2^
Cornea	RNA Mis-splicing in Fuchs Endothelial Corneal Dystrophy II	*H. sapiens*	GSE112201	TopHat2, edgeR	Wieben and colleagues^31^
Cornea	RNA Mis-splicing in Fuchs Endothelial Corneal Dystrophy	*H. sapiens*	GSE101872	TopHat2, edgeR	*
Cornea	Transcriptome profiling of human keratoconus corneas through RNA-sequencing identifies collagen synthesis disruption and downregulation of core elements of TGF-β, Hippo, and Wnt pathways	*H. sapiens*	GSE77938	Bowtie2, StringTie, Cufflinks2, Kallisto, DESeq2, edgeR	Kabza and colleagues^32^
Diabetic retinopathy	Transcriptomic Analysis of Endothelial Cells from Fibrovascular Membranes in Proliferative Diabetic Retinopathy	*H. sapiens*	GSE94019	Partek	*
Muller glia	Rapid, dynamic activation of Müller glial stem cell responses in zebrafish	*Danio rerio*	GSE86872	RSEM, edgeR, limma	Sifuentes and colleagues^33^
Retina	Id2a knockdown in zebrafish retina	*D. rerio*	GSE38786	Bowtie, DESeq, DAVID	Uribe and colleagues^34^
Retina	Molecular anatomy of the developing human retina	*H. sapiens*	GSE104827	STAR, RSEM, limma,	Hoshino and colleagues^35^
Retina	The Dynamic Epigenetic Landscape of the Retina During Development, Reprogramming, and Tumorigenesis	*H. sapiens*	GSE87042	TopHat2, Cufflins2	Aldiri and colleagues^36^
Retina	Unprecedented alternative splicing and 3 Mb of novel transcribed sequence leads to significant transcript diversity in the transcriptome of the human retina	*H. sapiens*	GSE40524	RUM pipeline	Farkas and colleagues^15^
Retina	Comparative Systems Pharmacology of HIF Stabilization in the Prevention of Retinopathy of Prematurity	*Mus musculus*	GSE74170	TopHat, Cufflinks	Hoppe and colleagues^37^
Retina/CRX	Graded Expression Changes Determine Phenotype Severity In Mouse Models of CRX-Associated Retinopathy	*M. musculus*	GSE65506	TopHat, edgeR	Ruzycki and colleagues^38^
Retina/Macula	Comprehensive analysis of gene expression in human retina and supporting tissues	*H. sapiens*	GSE94437	GSNAP, Cufflinks2	*
Retina/RP	rd10 transcriptome analysis	*M. musculus*	GSE56473	RMap, edgeR	Uren and colleagues^39^
Retina/RPE	Comprehensive analysis of gene expression in human retina and supporting tissues	*H. sapiens*	GSE94437	GSNAP, Cufflinks2	*
Retina/RPE	Region-specific Transcriptome Analysis of the Human Retina and RPE/Choroid	*H. sapiens*	PRJNA336370	TopHat2, Cufflink2, cummeRbund	Whitmore and colleagues^40^
Retina/RPE/ES	Comparative transcriptomic analysis of self-organized, in vitro generated optic tissues	*M. musculus*	GSE62432	TopHat2, Cufflinks2, edgeR	Andrabi and colleagues^41^
Retinoblastoma	A three-dimensional organoid model recapitulates tumorigenic aspects and drug responses of advanced human retinoblastoma	*H. sapiens*	GSE120710	Kallisto	Saengwimol and colleagues^42^
Retinal Culture/iPSC	Treatment Paradigms for Retinal and Macular Diseases Using 3-D Retina Cultures Derived From Human Reporter Pluripotent Stem Cell Lines	*H. sapiens*	GSE103826	Not reported	Kaewkhaw and colleagues^43^
Retinal Culture/iPSC	Transcriptome dynamics of developing photoreceptors in 3-D retina cultures recapitulates temporal sequence of human cone and rod differentiation revealing cell surface markers and gene networks	*H. sapiens*	GSE67645	Bowtie2, eXpress, edgeR, limma	Kaewkhaw and colleagues^44^
Retinal Culture/iPSC/ESC	Accelerated and Improved Differentiation of Retinal Organoids from Mouse Pluripotent Stem Cells in Rotating-Wall Bioreactors	*M. musculus*	GSE102727	edgeR, limma	DiStefano and colleagues^45^
RGC/ESC	Enriched retinal ganglion cells derived from human embryonic stem cells (RNA-seq)	*H. sapiens*	GSE84639	ExAtlas	Gill and colleagues^46^
RPE	Aneuploidy-induced cellular stresses limit autophagic degradation.	*H. sapiens*	GSE60570	RSEM, Bowtie, DESeq, ssGSEA	Santaguida and colleagues^47^
RPE	Regulation of protein translation during mitosis	*H. sapiens*	GSE67902	Bowtie, DAVID	Tanenbaum and colleagues^48^
RPE	RNA-Seq analysis of 4N and 2N RPE1 cells following polyploid induction via cytokinesis failure or Aurora kinase inhibition [tpo3]	*H. sapiens*	GSE86101	TopHat2, edgeR	Potapova and colleagues^49^
RPE	RNA-Seq analysis of proliferating 4N and 2N RPE1 cells derived from single cell clones following inhibition of Aurora B to induce polyploidization [tpo10]	*H. sapiens*	GSE86103	TopHat2, edgeR	Potapova and colleagues^49^
RPE	RNA-Seq analysis RPE1 cells following exposure to Nutlin-3 to identify target genes of p53 [tpo12]	*H. sapiens*	GSE86104	TopHat2, edgeR	Potapova and colleagues^49^
RPE	Appropriately Differentiated ARPE-19 Cells Regain a Native Phenotype and Similar Gene Expression Profile	*H. sapiens*	GSE88848	CLC Genomics Workbench, DESeq2	Samuel and colleagues^50^
RPE/AMD	Reversal of persistent wound-induced retinal pigmented epithelial-to-mesenchymal transition by the TGFb pathway inhibitor, A-83-01	*H. sapiens*	GSE67898	Partek, edgeR	Radeke and colleagues^51^
RPE/AMD	A widespread decrease of chromatin accessibility in age-related macular degeneration	*H. sapiens*	GSE99287	TopHat2, Cufflinks2	Wang and colleagues^52^
RPE/iPSC	Expression data for hiPSC-derived RPE treated with 10mM Nicotinamide or vehicle	*H. sapiens*	GSE90889	STAR, bedtools, samtools, DESeq2	Saini and colleagues^53^
RPE/iPSC	Comparison of stem-cell derived retinal pigment epithelia (RPE) with human fetal retina pigment epithelium	*H. sapiens*	GSE36695	Galaxy - TopHat2, Cufflinks2	*

AMD, age-related macular degeneration; DAVID, Database for Annotation, Visualization, and Integrated Discovery; iPSC, induced pluripotent stem cell; NCBI, National Center for Biotechnology Information; RGS, retinal ganglion cell; RNA-seq, RNA-sequencing; RP, retinal pigment; RPE, retinal pigment epithelia.

### RNA isolation

Within our cells, several RNA species are present at any one time serving differing roles. Through transcription of genes, there are protein-encoding mRNAs. Small RNAs involved in translation include transfer RNAs (tRNAs) and ribosomal RNAs (rRNAs). Regulatory RNA species, include antisense RNAs (asRNAs), microRNAs (miRNAs), Piwi-interacting RNAs (piRNAs), small interfering RNAs (siRNAs), short hairpin RNA (shRNA), and long noncoding RNA (lncRNA), all play a role in gene expression regulation. Highly abundant rRNA species, the predominant component of the ribosome involved in protein synthesis, constitutes up to 90% of the total RNA in cells. rRNA may require removal from samples to produce a library with considerably more representation of mRNA transcripts. Methods for rRNA removal include enriching mRNA using poly(A) selection, targeting the polyadenosine monophosphates at the 3′ tail of mature mRNA species, or depletion of rRNA by systems such as Ribo-Zero (Illumina, CA, USA) and duplex-specific nuclease degradation.^56^ rRNA depletion is an essential consideration for formalin-fixed and paraffin-embedded (FFPE) samples where RNAs are potentially degraded to a small average size, under 200 nucleotides.^57^ rRNA depletion should also be considered when the biological sample cannot provide enough quantity or high-quality mRNA through poly(A) selection.^58,59^ For samples with a small amount of starting material, there are specific library preparation systems available, such as SMART-seq (Takara Bio, CA, USA), relying on pre-amplification of fragments and may include a second stage of amplification.^60^ This can result in variable 3′ end bias representation of genes in library preparation, although the overall effect on expression values may be negligible.^61^ Small RNA species, such as those lacking poly(A) signals, can be assessed through small RNA-seq protocols.^62^

### Library preparation and platforms

To convert RNA into a library of molecules for sequencing, generally, it is first fragmented to an appropriate size for the chosen platform, either by physical or enzymatic approaches. First-strand complementary DNA (cDNA) is synthesized from the RNA sequences. Dependent on the platform and library kit used, platform-specific adapter sequences may be incorporated to the ends of the molecules to enable subsequent sequencing. Some systems add adapter sequences through ligation after cDNA synthesis (including Illumina TruSeq, Takara Clontech SMARTer, PerkinElmer NEXTflex, and KAPA Biosystems); other sequences may be attached to each molecule, including an inline index to identify the sample, allowing multiplexing of libraries when sequencing. Inline barcodes can be utilized to provide a label of origin for each RNA molecule.^63^ Recent developments include unique molecular identifiers (UMIs), molecular tags consisting of several random bases that can be used to detect and quantify unique transcripts.^63–65^ Unwanted duplication of reads through amplification methods can readily be detected.^66,67^ The addition of UMIs significantly improves the accuracy of gene quantification, especially high expressing genes.^68,69^ The resulting library of cDNA molecules can then be assessed for quality before sequencing.

For library preparation, one must consider how much RNA will be available for the experiment as well as the specific library types required, for example, those that maintain strand information or need significantly lower input RNA levels, such as those from FFPE or laser-captured micro-dissected samples,^70^ and single-cell RNA-seq (scRNA-seq).^71^ Strand-specific RNA-seq can resolve the ambiguity of overlapping genes transcribed on opposite strands, allowing identification of antisense expression by retaining information from which DNA strand the RNA was first transcribed.^72^ This information can be maintained using approaches that either incorporate a chemical modification in the second cDNA synthesis stage, with subsequent digestion of the nonmodified strand, or incorporate distinct primer adapters with the RNA.^73,74^ Library preparation protocols differ to achieve specific goals; TruSeq^TM^ (Illumina) is a general method chosen when starting material is not restricted; Smart-Seq2 and Ovation (NuGen, CA, USA) are suited to low input amounts.^60,70,75^

High-throughput sequencing approaches are rapidly evolving regarding both technology and chemistry. Illumina, PacBio RS, Oxford Nanopore, and Ion Torrent are some of the most commonly utilized platforms.^76,77^ The Illumina short read ‘sequence-by-synthesis’ systems have been rapidly adopted by the research community due to high data throughput, accuracy, availability, and declining costs.

### Sequencing

Sequencing depth, the number of fragments sequenced per sample, remains a critical factor for RNA-seq design. Studies have reported that increasing reads does not always provide increased biological significance.^16,17^ However, detection of lower abundance RNA species requires increased read sequencing, although RNA-seq shows a greater dynamic range than other assays.^78,79^ For the analysis of differential gene expression alone in human samples, 10–20 million reads per sample would provide significant information on most genes expressed. Investigation of alternatively spliced, novel isoforms, or fusion events, will require higher read number to capture the expression patterns, although increasing reads are associated with increased noise.^17^

With the depth of sequencing and library construction, comes the considerations of single-end reads or paired-end reads and read length. cDNA products may be sequenced from either single or both ends (paired). For simple differential expression analysis, single-end reads can provide valuable information. Paired-end reads, due to the size of RNA fragments produced (typically 300–500 nucleotides), will provide more significant information as the number of reads from fragments spanning exon–intron boundaries will be higher. As RNA-seq investigates transcribed and processed RNA, it is crucial that a level of aligned reads or paired-end fragments span exon boundaries. Single-end reads can be utilized for analysis of the 3′ regions of transcripts, such as with Tag-seq and MACE, assuming expression as a whole from sequencing the end region only.^80^

There are several biases in the analysis of RNA-seq differential expression: low-level transcripts producing high significance in expression-level differences and longer more abundant transcripts showing greater significance due to large number of reads per library aligned to their reference sequence. Read length is highly dependent on the application; for gene expression, profiling short reads (50–75 basepairs, bp) will detect the majority of RNA species in a library; for analysis of the transcriptome including identification of novel annotations, paired-end reads of 100+ bp will enable complete coverage of transcripts and novel splice sites; and for small RNA analysis, a read length of 50 bp would provide coverage of the majority of RNA due to their size. Long read sequencing is also possible using systems including PacBio and Oxford Nanopore, providing detailed analysis of specific isoforms expressed as well as allele-specific expression patterns, allowing the development of personalized transcriptomes.^81^

## RNA-seq analysis at the mRNA level

**Figure 2.**
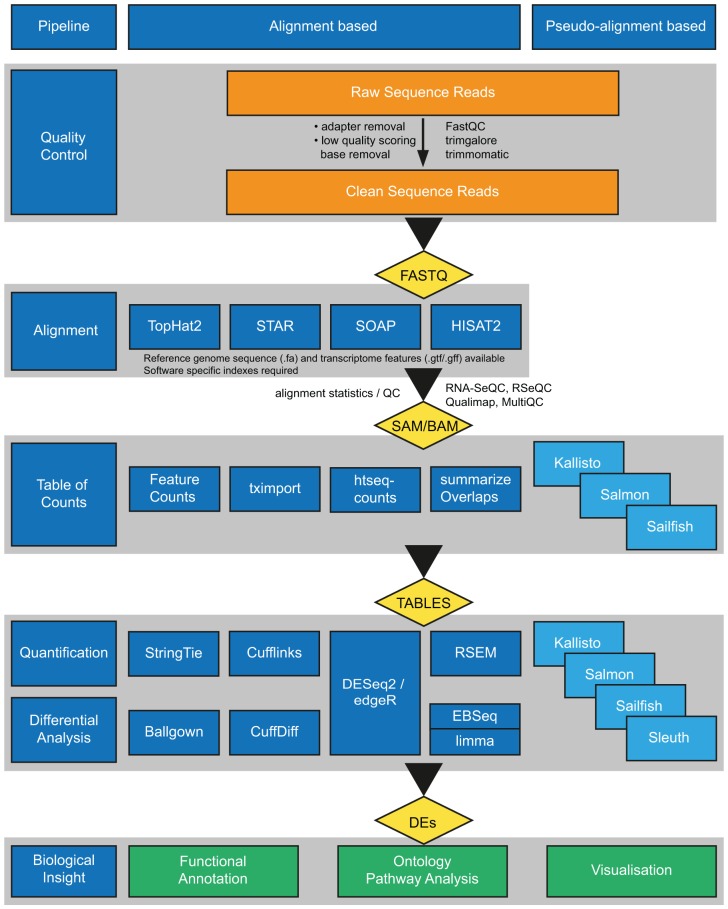
Schematic representation of typical bioinformatic processing of high-throughput sequence data for RNA-seq experiments. The sequencing platform generated raw reads (FASTQ) are subjected to quality assessment. Where a reference genome and a high-quality annotation are available, resulting high-quality cleaned reads can be used in alignment- or pseudo-alignment-based processes. For alignment-based process, reads are mapped to the genome and transcriptome in a splice-aware manner. Resulting alignments (SAM/BAM/CRAM) are assessed for mapping qualities and counts of features (genes/transcripts/exons) generated. Counts are modeled for quantification and differential analysis computed using various methods, resulting in differential feature lists. With pseudo-alignment-based methods, clean reads are modeled to the transcriptome, allowing direct quantification of appropriate feature(s) for differential analysis. The output of both approaches can provide further insight through gene ontology analysis (GSEA/GO ORA), pathway analysis (Panther, KEGG, DAVID), and visualized (IGV, GenomeBrowse, Bioconductor) for report production. Software examples listed are non-exhaustive. DAVID, Database for Annotation, Visualization, and Integrated Discovery; GO, gene ontology; GSEA, gene set enrichment analysis; IGV, Integrative Genomics Viewer; KEGG, Kyoto Encyclopedia of Genes and Genomes; ORA, over-representation analysis; Panther, Protein Analysis Through Evolutionary Relationships; SAM/BAM, sequence/binary alignment map.

Commonly, RNA-seq experiments investigating differential gene expression follow the stages outlined in Figure 2. Once sequencing data are generated, it requires alignment to either the genome or transcriptome reference sequences. In situations where novel transcripts are of interest, alignment to the genome followed by *de novo* transcript assembly is required. After alignment, feature counts are calculated and normalized, and differentially expressed features are identified. How these changes are biologically relevant to the experimental hypothesis is the final stage of investigation.

There are an increasing number of tools and methods of analysis for RNA-seq data sets, with each stage of the study requiring appropriate quality control. Aside from command-line tools and cloud-based approaches, commercial products include CLC Genomics Workbench (Qiagen, CA, USA), DNAnexus, Ingenuity IPA (Qiagen), and Partek Genomics Suite. Software for differential expression analysis has been evaluated using both experimental and simulated data sets. Comprehensive reports of such tools have been presented previously.^82–85^ Combination of approaches using different tools has led to improved results.^86^ Therefore, it is recommended to utilize multiple pipelines on the data set and understand fully the differences and similarities in the results. For this review, we will focus on several commonly cited, free, open-source tools to achieve differential expression analysis of human samples. The tools mentioned are not intended as an extensive list.

### Read quality control

The Illumina sequencing platform will produce raw FASTQ files that represent the sequence of the library in question. FASTQ is a text-based file format including all the sequence data along with associated quality scores. Each Phred score represents a log-scaled estimated probability of error in the base being called, for example, a score of 30 indicates a 1 in 1000 probability that the base is incorrect. Initial processing of these read files should include quality assessment of the base calls using tools such as FASTQC^87^ or FASTX-Toolkit.^88^ These provide graphical summaries of the sample reads, allowing quick visual identification of potential problems. Issues may commonly include over-represented sequences (e.g. adapter sequences or rRNA) or low-quality scoring bases at the 3′ end of reads. Tools to process the reads, filtering of poor bases, and trimming bases and adapters include Trimmomatic,^89^ Trim Galore,^90^ and cutadapt.^91^

### Read alignment

Post-processing of the cleaned FASTQ reads requires either alignment to the human genome, such as Ensembl GRCh38 or NCBI hg38 builds, or the associated human transcriptome or pseudo-alignment to the transcriptome and count modeling with tools such as Salmon,^92^ Kallisto,^93^ and Sailfish.^94^ Alignment of reads requires software that can process mapping in a splice-aware manner.^95,96^ Many reads generated will span splice junction coordinates, and alignment will require algorithms to split reads to different exonic positions. Mapping software includes HISAT2,^97^ SOAPsplice,^98^ TopHat2,^99^ and STAR.^100^ These produce a sequence/binary alignment map (SAM/BAM^101^) file of the reads aligned to the genome. Alignments may be visualized using tools such as Integrative Genomics Viewer (IGV)^102,103^ or GenomeBrowse,^104^ providing an insight to read metrics at the feature level. One of the greatest challenges is the subsequent assignment of aligned reads to transcripts they originate from to infer gene expression. Several new generation tools have introduced alignment-free transcript or gene quantification methods.^92–94^ These utilize *k*-mer-based matching to indexed transcript data sets, breaking reads into smaller *k*-mers, resulting in significantly faster analysis.^94^ A recent report, while confirming different pipeline performance was virtually identical for *in vivo* transcripts, demonstrated that alignment-based approaches were superior to alignment-free pipelines for total RNA analysis, as both small genes and low-expressed genes biased the accuracies of alignment-free approaches.^105^

### Read duplication

Post alignment, processing of the data includes sorting by genomic coordinates and marking reads that can be assigned as optical or polymerase chain reaction (PCR) duplicates,^106,107^ using tools such as Picard.^108^ There is significant discussion as to whether such reads should be removed from the analysis, as preferential amplification of cDNA fragments in the library preparation could result in a gene/isoform having an increased level of reported expression if such duplicated fragments were included.^109,110^ Other biases can consist of fragment GC-content, priming of reverse transcription by random hexamers, and rRNA depletion methods.^70,111,112^ A common practice to handle PCR duplicates would include removal of all but one representative read of identical sequences; however, this assumes that all identical reads were generated by PCR from the sample cDNA molecule.^113^ If removed, biologically significant information may be lost as smaller genes have reads that span the same genomic coordinates. UMIs enable tracking of fragments through library preparation, sequencing, and data analysis to overcome such biases.^66,114^

### Feature summarization

Expression levels of features, either at the gene or transcript level, are estimated from mapped read counts where appropriate feature annotation files exist. There is currently no consensus approach that is the most suitable to all situations, although this is an area of significant recent development.^85^ Initial analysis of RNA distribution through technical replicates fitted well to a Poisson distribution, in which reads map to the transcriptome in a random unrelated fashion within the library.^22,115,116^ With decreasing cost and speed, the use of higher numbers of biological replicates demonstrated that sample variability was greater than the expected distribution, giving increased false positives. Subsequent methods to handle this variability include analysis based on negative and beta-negative binomial statistical models, such as edgeR,^117^ BaySeq,^118^ Cufflinks2,^119,120^ and DESeq2.^121^ Each requires an input of sample counts per gene or transcript that can be created with tools such as featureCounts^122^ or htseq-count.^123^ Raw counts generated are not suitable for comparison of expression levels. Transcript length and library read size are primary factors creating bias in such data. This high-dimensional count data are, therefore, fitted to the model and normalized by the chosen package. There are several metrics for normalization of gene expression, including RPKM (reads per kilobase per million mapped reads),^124^ FPKM (fragments per kilobase per million mapped reads), and TPM (transcripts per kilobase million).^125^ RPKM and related FPKM for paired-end sequences normalize gene coverage through correction of differing sample sequencing depth and RNA length. However, RPKM has been shown to be a poor metric for RNA abundance between samples.^125^ RPKM is calculated by dividing the read counts per feature with a scaling factor (total number of reads in the sample x 10^−6^) and by the length of the gene (kb). Within the sample, the RPKM values can be assessed for comparative expression analysis; however, due to the nature of the variation of library sizes between samples, RPKM values will not be comparable, leading to confusion in the literature and the use of RPKM.^125^ TPM overcomes this by reordering the calculation, normalizing for gene length followed by normalization for library sequencing depth. Therefore, the sum of all TPMs in each sample will be the same, unlike RPKM/FPKM. This provides the opportunity for cross-sample expression-level comparisons. With the size of the dimensional data generated for gene counts, correction of statistical validity is required; a common approach is using false discovery rate (FDR) procedures to correct for multiple tests, for example, using the Benjamini–Hochberg method.^126^ Such processes are aimed at controlling the number of false positives when the null hypothesis has been incorrectly rejected.

### Differential gene analysis

One of the most commonly used protocols with RNA-seq data analysis is the assessment of changes in expression of genes between sample conditions. Several methods have been produced to normalize and model the count data produced from aligned short reads. Generally, the input for these tools will be raw counts, to avoid biases introduced through normalization. Methods to model expression from count data include DESeq2,^121^ Cufflinks2,^119^ NOISeq,^127^ and edgeR.^117^ When working with transcript-level features, a further consideration would be the change in transcript length across samples/conditions that would alter intra-sample calculations.^119^ Comparative analyses of techniques used for differential expression studies have been reported^85,116,128,129^ and reviewed.^84,130,131^ Differential expression analysis results in lists of differentially expressed genes (DEGs) or features and associated fold changes. Decision on biological significance to filter the data set relative to fold change and adjusted *p*-value thresholds is highly dependent on the experimental design, which usually will require manual interactive inspection of the data. Principal component analysis (PCA) reduces the data dimensionality down into components of variation.^132^ By taking the main components and plotting them in either two- or three-dimensional (2D or 3D) space, samples can be visualized to enhance interpretability. PC1 describes the prominent variation within the data, PC2 the second, and so on. This aids visualization of groupings between replicates as well as potentially identifying sample outliers.

Transcript-level differential expression analysis may assist in the detection of isoform changes. Transcripts can be assembled using tools including Cuffdiff2 or StringTie^133^ that assemble reads into potential transcripts, using prior knowledge, but also will identify novel transcript isoforms, followed by comparison of expression levels. Alternative splicing occurs in 90–95% of genes in mammals; therefore, analysis of the alternative use of exons and splice sites from RNA-seq data is of vital importance.^134,135^ Tools to assess differential usage of features such as exons or splice sites handle the information differently, using an exon-based approach, comparing to the overall expression of the associated gene.^136,137^ Each has potential benefits and drawbacks, summarized in a recent report on the assessment of tools available.^138^

Batch effects can be a significant source of variation between batches of samples, resulting in reports of false DEGs. There are a number of approaches to correct for known or unknown batch effects, including surrogate variable analysis (SVA)^54^ and ComBat.^139^ Numerous tools have been designed with batch effect correction stages optional and evaluated.^55^ Batch effects can ultimately range from increasing variability and reducing the power of an experiment, to becoming confounded with a desirable outcome and result in misleading biological interpretation.

### Data visualization

Visualization of RNA-seq data can be achieved in several ways, similar to other forms of high-throughput sequencing data. Genome browsers such as IGV, UCSC,^140^ and GenomeBrowse enable the user to view read alignments, highlighting read coverage and alternative splicing events with Sashimi plots, and summarizing the mapped read density over exons and junctions on the gene model.^141^ Combined with differential expression and differential usage data, display of individual genes of biological interest at the exon level can be used to assess potential complications from read alignment artifacts, for example, specific regions of the genome remain difficult to either sequence or align to with short read sequencing.^142,143^

Data exploration throughout the analysis pipeline ensures precise results being reported. Useful tools for summarizing data from raw or processed sequence reads as well as alignment statistics visually include MultiQC,^144^ QualiMap,^145^ and RNA-SeQC.^146^ This type of data visualization enables querying of read alignment efficiency as well as proportions mapped to features such as exons, introns, and splice sites.

### Biological insight

The biological significance of changes in the global transcriptome can be investigated through pathway enrichment of the list of DEGs/transcripts. Two example methods to aid functional significance assignment include (1) over-representation analysis (ORA), which compares the list of filtered DEGs against the annotated genome for over-represented functional assignment,^147^ and (2) gene set enrichment analysis (GSEA), which utilizes the complete data set, ranking the entire transcriptome according to the expression-level changes using differing metrics.^148^ Both rely heavily on prior knowledge and functional assignment to genes through Gene Ontology terms and databases such as MSigDB.^148^ Specific tools have been created for such analysis, which invariably demonstrates gene length bias, where larger genes have a greater chance of showing significant changes. GOSeq, a Bioconductor package, aims to estimate and account for such bias.^149^ Analytical tools continue to develop; PathwaySplice addresses explicitly bias through accounting for number of exons/junctions and performs pathway enrichment analysis.^150^ Functional annotation data can also be readily queried using DAVID (Database for Annotation, Visualization, and Integrated Discovery),^151^ Panther (Protein Analysis Through Evolutionary Relationships),^152^ QuickGO,^153^ and STRING.^154^ ClueGO, a Cytoscape app, enables rapid querying of ontology databases, producing clustered terms in a functional network.^155^ GSEA requires predefined collections of gene sets for analysis of the RNA-seq ranked list data set, including Kyoto Encyclopedia of Genes and Genomes (KEGG), Reactome, and BioCarta. GSEA provides a method for investigating changes in related sets of genes that may provide more insightful explanation than, for example, a large expression fold change of a single gene or numerous changes in genes with no biological theme. All genes detected experimentally are taken into consideration, not only those above the arbitrary cutoffs. Genes with small changes in expression that might not have reached the significance threshold may be of more biological importance within the same pathways, providing links between prior knowledge and newly generated experimental data.

Novel genes, as well as noncoding RNAs (ncRNA) identified in RNA-seq data sets, can present a challenge for functional ontology assignment. Protein sequence homology can be readily assessed for protein-coding transcripts using current databases. While no standard functional annotation route is defined for ncRNAs, databases such as miRbase,^156^ LNCipedia,^157^ and NONCODE^158^ maintain information on specific classes of ncRNA.

**Figure 3.**
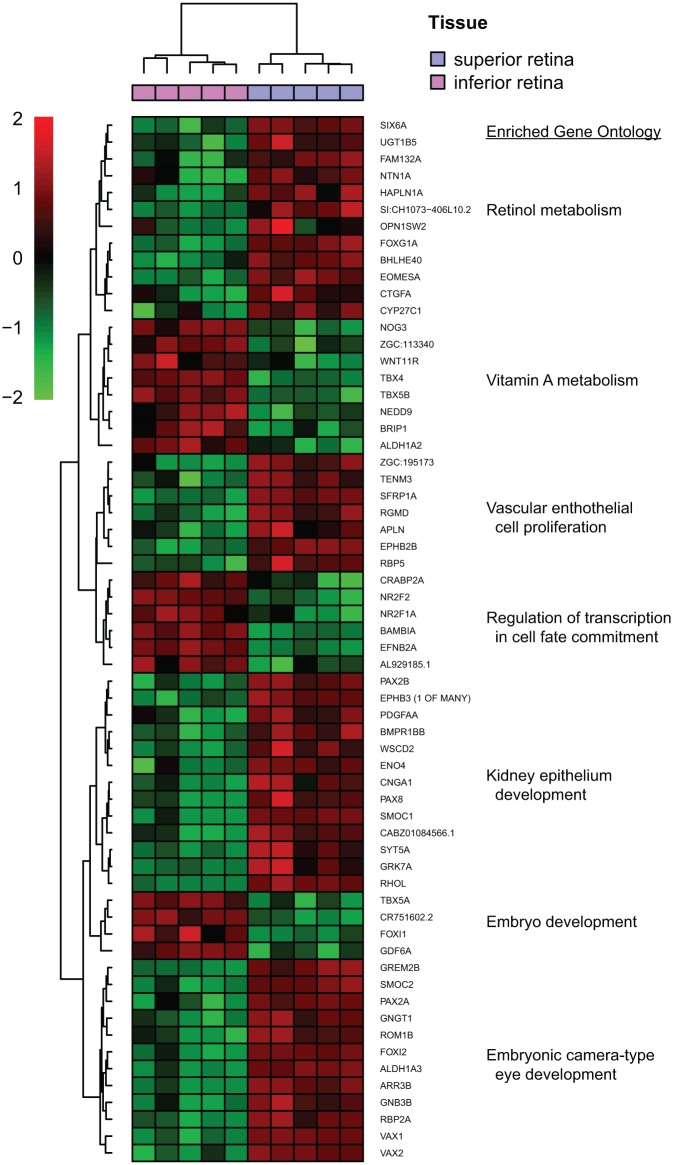
Heat map of differentially expressed genes (DEGs) in zebrafish between isolated optic fissure tissue and dorsal retina at 56 hours post fertilization (hpf),^159^ generated by R for Statistics package NMF. DEGs were identified using DESeq2, whose output was filtered for biologically significant results using criteria of a false discovery rate of less than 0.01 and fold change greater than 2. Resulting DESeq2 analysis was rlog transformed and hierarchical clustering performed on differential gene list. The z-score scale bar represents relative expression ±2SD from the mean. Top enriched gene ontology for biological process (BP) is highlighted for each cluster.

Highly similar ontologies cluster, highlighting the overall trends and themes of the underlying biological data. The expression of significant DEGs can be assessed through the generation of heat maps; a visualization method for rows of data, such as counts or expression values, related to the mean of that row. By calculating the *z*-score, the number of standard deviations from the mean expression of a gene, each sample’s expression can be represented through color variations. Hierarchical clustering, a way of arranging items in a hierarchy based upon similarity, can be used alongside heat maps to produce a dendrogram that shows the relationship between the rows [in this example, genes differentially expressed during zebrafish optic fissure fusion^159^ (Figure 3)]. One-way cluster analysis will identify clustering based upon similarity of abundant data in one dimension, such as expression patterns of genes (row) for example, whereas two-way clustering will also cluster on the second data dimension, for example, similarity of the sample profiles (column) commonly using Euclidean distances.^160^ The aim is to identify subsets of genes in samples so that when one data dimension (gene) is used to cluster another dimension (sample), clear and significant partitions emerge.

### Reproducibility

Throughout the analysis of any data set, it is critical to maintain reproducible workflows, providing detailed information on how data are manipulated, filtered, and assessed.^161,162^ Even so far as versions and dates of databases utilized are critical to maintain the integrity of the results. There are diverse approaches to maintaining reusable and reproducible bioinformatics pipelines such as Subversion and Git (this provides a version control system, preserving the history of the document). GitHub provides an open-source online resource for project tracking, sharing, and issue discussion. Code can also be created, shared, and annotated similarly with Jupyter scientific computing notebooks. Other options for reproducible analysis include AWS Elastic Cloud Computing,^163^ Docker,^164^ and Galaxy.^165^ Galaxy provides a web-based platform for high-throughput sequence data analysis. This platform is accessible to users without programming experience by providing a graphical web interface to command-line tools as well as predefined shared workflows and parameters. Tools and pipelines continue to develop rapidly with Galaxy adopting many of these improvements.^166,167^

## Utility of RNA-seq in ophthalmology research

Vision research has benefited significantly from the use of RNA-seq over recent years.^168^ Characterization of human diseases related to the eye can prove difficult due to the lack of high-quality human tissue required for the analysis; therefore, model systems, such as animals or cell-based, provide vital resources to further our understanding of eye development and disease. The role of noncoding and circular RNAs in eye disease has been the subject of a recent review.^169^ Here, follow some applications in ophthalmology and vision research.

### Human retina

Transcriptome analysis of three human donor adult healthy eyes has provided insight into which RNAs are expressed specifically in human retinal tissue. Farkas and colleagues identified 79,915 novel alternative splicing events that included 29,887 novel exons and 28,271 novel exon skipping events with 116 potential novel genes expressed in retina. The observations, while highly reproducible, indicate a high level of novelty in the makeup of the retinal transcriptome that highlights the difference between species and the importance of characterization of human tissue.^15^ Further comprehensive analysis of eight normal eyes has been carried out, demonstrating transcriptome differences between macular and peripheral retina.^170^ Approximately, 80% of the annotated transcriptome was reported to be expressed in the retina, which showed significantly different alternative splicing patterns to the RPE, choroid, and sclera; hence, spatiotemporal gene localization is needed. Analysis of mature mRNAs and ncRNA such as long-intervening ncRNAs (lincRNAs) has been shown to be involved in numerous cellular pathways in development and disease. Analysis of total RNA within both fetal RPE and iPSC-derived RPE identified over 1000 lincRNAs and 180 novel genes expressed in fetal RPE. The research also confirmed that the transcriptomes of iPSC-RPE were comparable to fetal RPE, so enforcing the suitability of these cells for vision research.^171^

While global transcriptome analysis via RNA-seq has fueled our understanding of underlying mechanisms of disease, ultimately it provides little information on the basic unit of biology, the cell. Since the development of scRNA-seq using in-house approaches, the field has seen an increase in the number of commercial options available. Protocols generally involve tissue disruption, which can lead to changes in expression profiles, although *in vivo* methods of mRNA isolation from tissue and prefiltering of cells based upon morphology and function have been produced.^172–174^ Post hoc PCA or hierarchical clustering of single-cell data has been relied upon to determine cell type classification. Recent scRNA-seq has identified up to 40 cell types of RGCs in the mouse retina using such approaches.^175^ While elucidation of model system RGCs has been invaluable, the need for further characterization of human cell types remains vital. To address this, human pluripotent stem–derived RGCs were profiled using scRNA-seq, showing a variable expression pattern of common RGC-associated genes, further indicating diversity within the cell population.^176^

### Retinal dystrophies

Currently, mutations in over 75 genes can cause retinitis pigmentosa (RP), affecting the RPE and or photoreceptor cells, leading to progressive loss of vision. Stem cell–based therapies offer potential treatment avenues, either replacement of retinal cell types through differentiation protocols or protection via general neuronal lineage cells.^177^ Using a rat model of progressive photoreceptor degeneration harboring a mutation in the *Mertk* gene (Royal College of Surgeons, RCS rat), RNA-seq has been used to elucidate expression changes post stem-cell transplantation with human neural progenitor cells (hNPCs).^178^ Comparative analysis of gene expression profiles of treated and untreated RCS rats and controls identified 68 genes with altered expression patterns due to treatment with hNPCs. Pathway analysis revealed an enrichment of signaling involved in phagocytic response alongside the increase in photoreceptor cell survival. The underlying *Mertk* mutation causes improper phagocytosis of photoreceptor outer segments, and restoration of phagocytosis by hNPCs is encouraging. Similarly, mouse models of RP, including the *rd10* mouse harboring a mutation in *Pde6b*, have been used to assess transcriptional changes underlying photoreceptor degeneration.^39^ Decreased expression of rod-specific genes was associated with a clear increase in Muller-specific gene expression, although other cell type-specific genes were dysregulated.^39^ Interestingly, alternative splicing of 284 genes was altered in the degenerated retina, with predominantly increased exon inclusion.

### Age-related macular degeneration

Understanding the pathogenesis of age-related macular degeneration (AMD) has been challenging due to the multifactorial etiology.^179^ AMD is characterized by RPE degeneration and consequent photoreceptor cell death. Although implicated in AMD, RPE phagocytosis has only recently been demonstrated to be dysfunctional by transcriptome analysis of RPE cells isolated from post-mortem AMD and normal age-matched control human eyes.^180^ To explore the disease progression, rat models of AMD were assessed for temporal changes in retinal transcriptomes. Enrichment ontology analysis has provided insight into cellular differentiation and developmental processes, all differential expression events were downregulated in comparison to controls. Gene clusters identified differing gene sets at the various disease stages linked to apoptosis.^181^ Targeted treatment of the exudative form of AMD through inhibition of vascular endothelial growth factor (VEGF) signaling using ascorbate-based targeted DNA hydroxymethylation has been validated via characterization of the resultant transcriptome in RPE cells (human fetal, rat and cell line ARPE-19) showing significant reduction in VEGF expression.^182^

### Corneal dystrophies

Corneal dystrophies are a group of genetic conditions that result in sight loss from various patterns of corneal opacity.^183^ Posterior polymorphous corneal dystrophy (PPCD) is a rare autosomal dominant disorder characterized by changes in Descemet membrane and the endothelial cell layer leading to decreased vision secondary to corneal edema.^183^ Although mutations in transcription factors *OVOL2* (type 1) and *ZEB1* (type 3) account for approximately 40% of all PPCD cases, the transcriptomes of PPCD endothelium and cultured human primary corneal endothelial cells were assessed to further elucidate potential biomarkers.^184,185^ Characterization of DEGs associated with *ZEB1* and *OVOL2* identified additional genes involved in proliferation, cell adhesion and migration, and cell morphology, which can be used to identify candidate genes for genetically unresolved patients.

### Glaucoma

Success in glaucoma treatment can be determined by the level of fibrotic encapsulation post trabeculectomy surgery.^186^ To further understand the fibrotic response, RNA-seq has been used to identify dysregulated genes between primary fibrotic and nonfibrotic fibroblast cell lines isolated from glaucoma patients.^187^ Genes involved in inflammation and apoptosis were significantly upregulated in the fibrotic cell type, including *RELB, PPP1R13L. MYOCD* (a critical cofactor of serum response factor regulating smooth muscle cell differentiation). *PRG4* was upregulated in nonfibrotic cells and has been associated with high levels of hyaluronic acid and scar-less fetal wound healing.^188^ In total, 246 genes were differentially expressed in fibrotic cell lines compared to nonfibrotic, providing an insight to a distinct fibrosis gene signature.

## Prospects

RNA-seq is now becoming the standard method of transcriptome analysis as both the tools and technology continue to develop. Methods of analysis differ significantly and validation of results using different tools remains uncertain. As more comparative studies are evolving, more appropriate use of tools will be forthcoming. Continued development of RNA-seq technologies has resulted in the ability to analyze minimal amounts of starting material, even from older fixed and embedded archived tissue. Development of single-cell techniques continues to be a highly dynamic area of research.^189–191^ Elucidation of cellular transcriptomes, in tissue-related context, will provide an insight into the regulation of gene expression in assumed identical cell types. Combined with temporal experimental designs, analysis of thousands of cells at a time, using techniques such as DROP-seq and InDrops, can provide detailed analysis of cellular subgroups within systems of interest.^192,193^ Recent adaption of scRNA-seq has allowed the reconstruction of cell lineage histories in model systems.^194–198^ Such large-scale informatics will drive knowledge of RNA expression through developmental stages and tissue types as well as providing the technology to approach many disease-related issues.

With the availability of open-access sequence data in online repositories including NCBI SRA and EMBL ENA, combined with the increase in computing power, increasing the speed of pipeline analysis, the amount of knowledge to be gained from transcriptome analysis is increasing. Combined with other ‘omics data, RNA-seq analysis has the potential to link gene expression with genomic features such as epigenetic changes, DNA sequence alterations, and protein interactions. The Department of Health and Social Care’s 100,000 Genomes Project, whose aim was to sequence 75,000 genomes of patients with rare diseases and cancer,^199,200^ concomitantly collected RNA alongside the DNA samples. This initiative will result in increased diagnostic rates and the discovery of novel disease-causing variants, while also providing an extensive wealth of information on transcriptomes from individuals with varied genetic backgrounds. For eye disease, the transcriptome will provide insights into how genes alter the development or function of the eye and has the potential to provide researchers with novel targets for therapeutic strategies.
